# Evolutionary characteristics and influencing factors of non-grain of cultivated land in main grain-producing areas—A case study of Lianyungang City, China

**DOI:** 10.1371/journal.pone.0325259

**Published:** 2025-06-11

**Authors:** Yunxia Zhang, Minne Liu, Xinsong Sun

**Affiliations:** 1 Lianyungang Real Estate Transaction and Registration Centre, Lianyungang, China; 2 School of Geography Science, Nanjing Normal University, Nanjing, China; 3 Jiangsu Dongtu Urban and Rural Planning and Design Co., Ltd. Lianyungang Branch, Lianyungang, China; Balochistan University of Information Technology Engineering and Management Sciences, PAKISTAN

## Abstract

It is significant to explore the evolution pattern and driving mechanism of non-grain of cultivated land in the main grain-producing areas to promote the sustainable development of agriculture and guarantee national food security. Taking Lianyungang City in Jiangsu Province as an example, the study uses remote sensing image interpretation, spatial autocorrelation analysis, and geographically weighted regression model to analyze the spatial and temporal evolution characteristics of non-grain of cultivated land, reveal the driving mechanism and formulate zonal regulation strategies. The results show that: (1) the level of non-grain of cultivated land in Lianyungang City increased gradually from 6.01% to 11.10% from 2002 to 2022, and grain cultivation was mainly shifted to greenhouse vegetables, construction and development and abandonment. (2) the level of non-grain of cultivated land showed a spatial pattern of high along the north-west-south-east and decreasing to the two sides, and the pattern showed a trend of gradual weakening, with Moran’s I decreased from 0.90 to 0.42. (3) The dominant factors of the spatial differentiation of non-grain of cultivated land in different periods are different, among which GDP, population density, NDVI, and precipitation are always the main influencing factors. The evolution of non-grain of cultivated land is a complex result of the joint action of resource endowment of farm households, location conditions, and economic policies. (4) The evolution of non-grain of cultivated land can be classified into single-factor-dominated, two-factor-dominated, and multifactorial effects. Three primary zones and differentiated zoning regulation strategies are proposed from three perspectives: subject synergy, government regulation, and system element enhancement. The study can provide a reference basis for promoting the protection and use of cultivated land and formulating differentiated agricultural management strategies in grain-producing areas similar to Lianyungang City.

## 1 Introduction

Food security is essential for stable economic and social development and national security. However, in the external context of climatic disasters [1], economic recession [2], intensification of localized conflicts [3], a global pandemic of the new coronavirus epidemic [4], uncertainty and instability in the supply of international agricultural markets have increased [5], and the importance and urgency of food security have grown [6,7]. The Global Food Crisis Report states that the number of people facing severe food insecurity globally has increased for the fourth consecutive year in 2022, with more than 250 million people facing severe hunger [8]. Domestic and foreign scholars generally agree that the non-grain of cultivated land is an essential reason for the above phenomenon [9–11]. In the case of China, although a strict use control system for cultivated land has been implemented, the problem of its non-grain use is still severe. The data of the third national land survey show that the cultivated land in the country has decreased by 7.52 × 10^4^km^2^ from 2009 to 2020, the net flow of cultivated land to forest land is about 7.47 × 10^4^km^2^, the net flow to garden land is about 4.20 × 10^4^km^2^, and the rate of non-grain continues to grow [12]. The reduction in the amount of cultivated land and the area sown for food profoundly affects the level of regional food supply. Therefore, exploring the evolutionary characteristics of the non-grain of cultivated land and its zoning control research is of great significance to promoting the sustainable use of cultivated land and guaranteeing national food security.

Currently, research on the non-grain of cultivated land has achieved milestones. Conceptually, domestic studies have divided the connotation of non-grain of cultivated land into two categories: narrow and broad, in which the narrow sense of cultivated land non-grain refers to the fact that cultivated land planted with food crops is no longer planted with food crops, focusing more on the change of growing crops at the plot scale. Cultivated land non-grain refers to the regional decline in the sown area and proportion of food crops [13], focusing mainly on the evolution of planting structure at the regional scale [14]. Foreign studies have concluded that most of the non-grain of cultivated land is caused by farmers not cultivating food [15,16], which is more similar to the narrow connotation of the non-grain of cultivated land in the country. The non-grain rate usually characterizes the level of cultivated land non-grain, the ratio of regional sown area of non-grain crops to the total sown area of crops [17,18], which is mainly calculated by using economic statistics [19] or field research data [20], or quantitatively analyzed by combining methods such as remote sensing [21], GIS technology [22], and model simulation [23]. Existing studies have mostly used regression models [24,25], multiple linear regression [26,27], and other methods to explore the influencing factors of the non-grain of cultivated land. Domestic studies believe that regional economic conditions, policy and regulatory constraints, and farmers’ willingness to grow food are the main driving factors of cultivated land non-grain [28–30], and the spatial heterogeneity of cultivated land non-grain in regions with different levels of economic development is differently driven by the intensity of the roles of economic efficiency drive and the lack of agricultural labor [31]. Foreign studies believe that the causes of agricultural land abandonment mainly include poor environmental conditions [32], low and unstable farm viability [33], poor production conditions [34], and inadequate management of natural resources [35]. Meanwhile, the existing research scales mainly focus on the national [14], provincial, and municipal macro-territories [19], and less on the micro-plot scale. On the whole, the existing research on non-grain of cultivated land is relatively rich in content, providing practical guidance for regional agricultural production and management of cultivated land. Still, there is a lack of research on spatial evolution analysis of different types of non-grain and zoning optimization governance carried out at the micro-plot scale, and the spatial variability of the impact of influencing factors on the non-grain of cultivated land, which makes it challenging to satisfy the requirements of fine control of agricultural production.

The Ministry of Natural Resources, the Ministry of Agriculture and Rural Development, and the State Forestry and Grassland Administration issued the Circular on Issues Related to the Strict Control of Cultivated Land Usage (Natural Resources Development [2021] No. 166) in 2021, which stipulates an “in-and-out balance” system of cultivated land to strictly control the conversion of cultivated land into other agricultural land such as forest land, garden land, and grassland. The main grain-producing areas, rich in cultivated land resources, are the lifeblood of national grain production. China’s main grain-producing areas contributed about 78% of grain production and 80% of commercial grain in 2022 [36], playing an essential role in grain supply [37]. Therefore, researching the non-grain of cultivated land in main grain-producing areas has become a pivotal path to strengthen the use control of cultivated land, which is of great practical significance for managing non-grain of cultivated land and enhancing regional grain production capacity. Given this, this study takes Lianyungang City, which is located in the Huaihai Economic Zone, China’s main grain-producing area, as an example and measures the level of cultivated land non-grain based on the explicit cultivated land non-grain data decoded from high-resolution remote sensing imagery from 2002 to 2022, analyses its spatial and temporal evolution, and then identifies the drivers of spatial evolution of non-grain with the help of the ordinary least-squares regression model and the geographically weighted regression model, and finally delineated the control area of non-grain of cultivated land and put forward the zoning control strategy. This study provides a theoretical basis for implementing a control system for cultivated land in major grain-producing areas.

## 2 Study area and research methods

### 2.1 Study area

**Fig 1:**
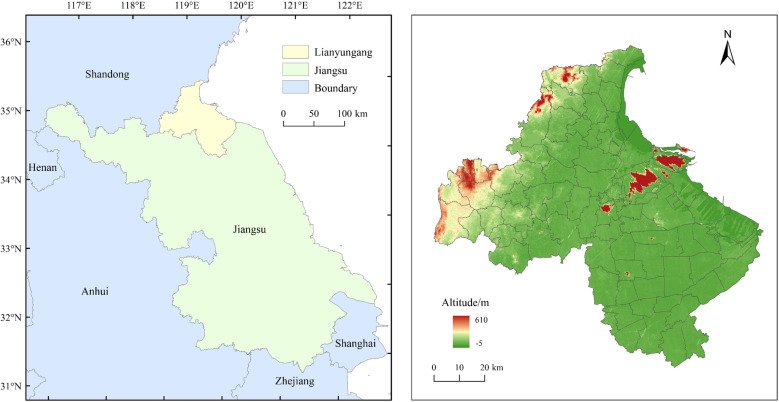
Location of the study area.

Lianyungang City (33°58′55″ ~ 35°08′30″N, 118°24′03″ ~ 119°54′51″E) is located in the northeastern part of Jiangsu Province, the total land area is 7,615 km² (Fig 1). It is separated from North Korea, South Korea, and Japan by the Yellow Sea in the east, connected to Huai’an City and Yancheng City of Jiangsu Province in the south, Xuzhou City and Suqian City of Jiangsu Province in the west, and adjacent to Rizhao City and Linyi City of Shandong Province from the north to the northwest. The landform includes the western Gangling area, the central plain area, the eastern coastal area, and the Yuntai Mountain area, with the terrain tilting from northwest to southeast. The city has a temperate monsoon climate with abundant rainfall in summer and scarce rain and snow in winter, which is favorable for the growth and development of crops. Lianyungang City had a grain sown area of 5,124.53 km² in 2022, with a total grain output of 3.68 × 10^6^t. The plantation industry is dominated by rice, wheat, cotton, soya beans, and peanuts, which occupy an important position in China’s production of agricultural by-products. The city has three municipal districts, namely Haizhou District, Lianyun District, and Ganyu District, and three county-level administrative districts, namely Gunnan County, Donghai County, and Ganyun County, with resident population of 4.60 × 10^6^ and an urbanization rate of 63.08%.

Lianyungang City is rich in agricultural resources and is a large city of food production. However, Lianyungang City has experienced significant climate change in recent years, with droughts breaking out [38], and with the advancement of urbanization and industrialization, the pressure on agricultural production is also surging, which poses a threat to agricultural production structure and food supply [39]. For one thing, to promote the development of rural industry, the local government encourages farmers to plant cash crops after the transfer of cultivated land, thus crowding out the area sown with food [40]; for another, with the intensification of the non-grain of the agricultural population, the scale of cultivated land planting and management in some areas has shrunk, leading to a decline in cultivated land yields, and the phenomenon of non-grain of cultivated land is increasingly prominent [41]. Therefore, choosing it as a research area can better reflect the typical characteristics of cultivated land non-grain, reveal its causes and impact mechanisms, and is of great significance to promote the protection of cultivated land and the sustainable development of agriculture for Lianyungang City, and can also provide useful reference and inspiration for other grain producing areas similar to Lianyungang City, and also has a more important demonstration significance for the promotion of the national management of the cultivated land non-grain.

### 2.2 Data sources and processing

The multi-source data mainly include remote sensing image data, socio-economic statistics, and geospatial data in this study, and the data sources and descriptions are detailed in Table 1. Among them, the plot data of cultivated land non-grain are based on high-resolution satellite images of 2002, 2012, and 2022, and all of them have a spatial resolution of better than 3 meters and have been pre-processed by alignment and geometric refinement correction. Due to the variety of data source types, the image data are archived historical aerial photographs with high spatial resolution but poor accuracy using automatic computer interpretation. Therefore, to ensure the extraction accuracy of cultivated land non-grain data, this study used ArcGIS10.8 software and manual visual interpretation method to divide the cultivated land non-grain into five types through land class comparison, namely, greenhouse vegetable planting, forest planting, aquaculture, non-agricultural construction occupancy, and abandonment of wasteland and verified the data of cultivated land non-grain data to ensure that the accuracy of the data reaches more than 90%.

**Table 1:** Data source and description.

Data name	Source	Resolution	Instruction
Land use change survey data	Relevant land authorities	–	Acquisition of information on various land types, such as cultivated land, rural settlements, roads, water bodies, etc., and polygons related to non-grain.
Various planning data	Relevant land authorities	–	Obtaining planning data on permanent basic farmland, high-standard farmland, functional food production zones, ecological red lines, etc.
Google Earth satellite image	Google Earth	3m	Extraction of non-grain polygons of cultivated land
DEM data	CAS Geospatial Data Cloud Platform	30m	Used to calculate altitude, slope, and other information
NDVI data	CAS Geospatial Data Cloud Platform	1000m	Used to calculate information such as vegetation cover
Meteorological data	China Meteorological Data Network (CMDN)	1000m	Obtaining data on precipitation, air temperature, wind speed, solar radiation, etc.
Economic and social data	CAS Geospatial Data Cloud Platform	1000m	Access to GDP, population density, transport network, etc.

### 2.3 Research methods

#### 2.3.1 Measurement of cultivated land non-grain.

Considering the identifiability of cultivated non-grain on remote sensing images and the impacts of different non-grain types, the study combined the land use structure of Lianyungang City [42,43] and the results of the field survey, classified the types of cultivated non-grain into five types, namely, greenhouse vegetable planting, forest planting, aquaculture, non-agricultural construction occupancy, and abandonment of wasteland. The cultivated land non-grain rate characterized the level of cultivated land non-grain. The calculation formula is as follows [44,45]:


NgA=Sv+Sf+Sa+Sc+Sd

NgR=NgA/St×100%

Where: *NgA* indicates the area of cultivated land non-grain, km^2^; *Sv* indicates the area of cultivated land that is occupied by greenhouse vegetable cultivation, km^2^; *Sf* indicates the area of cultivated land that is occupied by forest cultivation, km^2^; *Sa* indicates the area of cultivated land that is occupied by aquaculture, km^2^; *Sc* indicates the area of cultivated land that is occupied by non-agricultural constructions, km^2^; *Sd* indicates the area of cultivated land that is left fallow, km^2^; *NgR* indicates the rate of cultivated land non-grain, %; and *St* indicates the total area of cultivated land, km^2^.

Referring to previous studies (Xu et al., 2024; Chen, 20232), the study further classified the level of cultivated land non-grain into five levels by the natural breakpoint method, including lower non-grain (*NgR* ≤ 10%), low non-grain (10%< *NgR* ≤ 30%), moderate non-grain (30%< *NgR* ≤ 55%), high non-grain (55%< *NgR* ≤ 80%), and higher non-grain (*NgR*> 80%).

#### 2.3.2 Spatial autocorrelation analysis.

Each evaluation unit’s overall spatial aggregation characteristics are analyzed based on the domain-wide Moran index I, reflecting the degree of spatial correlation of the attribute values of spatially proximate units in the whole, and its value domain is [−1, 1]. $Moran^{\prime }sI\;>0$ indicates spatial positive correlation; the larger its value, the more apparent spatial correlation; $Moran^{\prime }sI\;<0$ indicates spatial negative correlation; the smaller its value, the larger spatial difference; in addition, $Moran^{\prime }sI\;=0$ suggests that the spatial distribution is random. The calculation formula is as follows [46]:


$$I=\frac{n}{\sum {\mathrm{\backslash nolimits}}_{i=1}^{n}\sum {\mathrm{\backslash nolimits}}_{j=1}^{n}W_{ij}}\times \frac{\sum {\mathrm{\backslash nolimits}}_{i=1}^{n}\sum {\mathrm{\backslash nolimits}}_{j=1}^{n}W_{ij}(x_{i}-\bar{x})(x_{j}-\bar{x})}{\sum {\mathrm{\backslash nolimits}}_{i=1}^{n}{\left(x_{i}-\bar{x}\right)}^{2}}$$

Local spatial autocorrelation indicates the degree of association between the attribute values of each spatial unit and its neighboring spatial units. The calculation formula is as follows [47]:


Ii=(xi−x¯)∑\nolimitsj[Wij(xi−x¯)]∑j(xi−x¯)2/∑j(xi−x¯)2n\nulldelimiterspacen

Where: $x_{i}$, $x_{j}$ is the level of cultivated land non-grain in regions i and j, respectively; $\bar{x}$ is the average value of the level of cultivated land non-grain in each evaluation unit; $W_{ij}$ is the spatial weight matrix (adjacency of spatial units), which $W_{ij}$ is 1 if regions i and j are adjacent to each other, and $W_{ij}$ is 0 otherwise.

The spatial aggregation of cultivated land non-grain includes four types: high-high, low-low, high-low, and low-high, where high-high and low-low indicate the spatial agglomeration and spillover effects with high and low levels of cultivated land non-grain, respectively, and low-high represents an outlier in which areas with low values of cultivated land non-grain are surrounded by areas with high values, and high-low represents an outlier in which areas with low values surround areas with high values of cultivated land non-grain.

#### 2.3.3 Ordinary least squares regression model(OLS).

Ordinary Least Squares linear regression model was used to assess the global relationship between the level of cultivated land non-grain and the driving factors with the following formula [48]:


$$y=\beta_{0}+\sum_{i=1}^{p}\beta_{i}x_{i}+\varepsilon$$

Where: y is the dependent variable, $x_{i}$ is the explanatory variable, p is the number of predictor variables, $\beta_{0}$ is the intercept, $\beta_{i}$ is the regression coefficient, and ε is the error term with mean zero and variance $\sigma^{2}$. The level of cultivated land non-grain was pre-transformed by Ln(X + 1), and the explanatory variables were standardized by Z. Stepwise multiple regression was used to select significant explanatory variables for the model. The best OLS model for the cultivated land non-grain was selected according to the following criteria: (1) the model’s adjusted R² was the highest; (2) the parameters of the model and the explanatory variables were significant (P ≤ 0.05); (3) the Variance Inflation Factor (VIF) of the explanatory variables was less than three.

#### 2.3.4 Geographically weighted regression model.

Since the random distribution of variables does not have independent spatial characteristics in the least squares model, a high degree of mutual independence between regions is required. Geographically weighted regression (GWR), on the other hand, introduces the spatial attributes of the data and detects the heterogeneity of spatial data through the function of geographic space having different spatial relationships and regional parameter estimation of spatial dependencies, which is formulated as follows [49,50]:


$$y_{j}=\beta_{0}(u_{j},v_{j})+\sum_{i=1}^{p}\beta_{i}(u_{j},v_{j})x_{ij}+\varepsilon_{j}$$

Where: $\left(u_{j},v_{j}\right)$ is the coordinates of the j raster; $\beta_{i}(u_{j},v_{j})$ is thei regression coefficient on the j raster, a function of geographic location; and $\varepsilon_{j}$ is the error term. This study identifies the optimal number of adaptive neighborhood points as adaptive bandwidth measured by AICc (Modified Akaike Information Criterion). Smaller bandwidths are used in data-dense places and larger bandwidths are used in data-sparse places. To avoid the effect of data covariance, the best stepwise OLS model was used to select the significant explanatory variables to be input into the GWR model. In this study, the dominant factors affecting the regional level of cultivated land non-grain were determined based on the results of the GWR model, and the zoning of dominant factors was carried out based on these results.

### 2.4 Analytical framework

**Fig 2:**
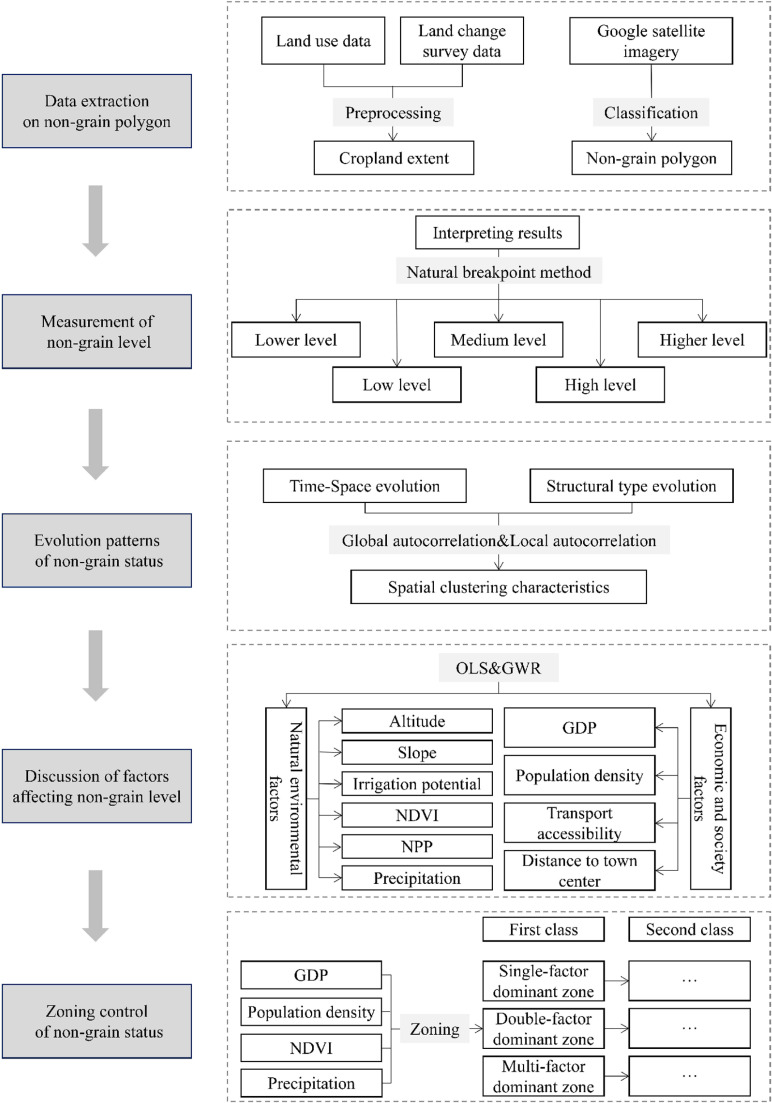
Analytical framework.

The analysis framework of this study is shown in Fig 2. Firstly, based on Lianyungang City land use data and change survey data, data pre-processing is carried out to extract the range of cultivated land in 2002, 2012, and 2022 as a mask for subsequent data classification and processing and selected Google Earth satellite images and the use of visual interpretation methods to carry out the extraction and classification of cultivated land non-grain plots. Secondly, based on the cultivated land non-grain interpretation data, measuring the level of cultivated land non-grain, and through the ArcGIS software using the natural breakpoints method of the cultivated land non-grain level is divided into lower, low, medium, high, and higher level of non-grain. Thirdly, analyze the spatial and temporal evolution of the non-grain level of cultivated land and the structural evolution of non-grain types, and based on this, use global spatial autocorrelation and local spatial autocorrelation to explore the spatial clustering characteristics of cultivated land non-grain. Finally, adopt OLS and GWR regression models, and select ten factors from natural and socio-economic factors, such as elevation, slope, GDP, and population density, to explore the influence of the factors on non-grain. Further selecting four factors that have a more significant impact on the evolution of cultivated land non-grain, namely, GDP, population density, NDVI, and precipitation, and zoning them according to their single-factor, two-factor, and multi-factor effects on the evolution of cultivated land non-grain, to reveal the driving mechanism of the influencing factors on cultivated land non-grain and to propose a differentiated zoning control strategy.

## 3 Analysis of results

### 3.1 Evolution of cultivated land non-grain in Lianyungang City

#### 3.1.1 Characteristics of spatial and temporal evolution of cultivated land non-grain.

The level of cultivated land non-grain increased significantly from 2002 to 2022 in Lianyungang City, with an overall increase of 5.09% in the non-grain rate (Table 2). Among them, the non-grain rate of cultivated land increased from 6.01% to 9.88% from 2002 to 2012, and the non-grain rate of cultivated land increased to 11.10% from 2012 to 2022. The area of lower and low degrees of non-grain increased by 2.34 percent and 0.31 percent, respectively; the area of medium degree of non-grain increased by 0.16 percent, the area of high degree of non-grain increased by 0.21 percent, and the area of higher degree of non-grain decreased by 3.01 percent.

**Table 2:** Temporal evolution of cultivated land non-grain in Lianyungang City from 2002 to 2022(%).

Year	Cultivated land non-grain rate				
	Lower level of non-grain	Low level of non-grain	Medium level of non-grain	High level of non-grain	Higher level of non-grain
2002	92.28	1.07	1.11	0.85	4.70
2012	92.49	1.11	1.11	0.92	4.37
2022	94.83	1.42	1.27	1.13	1.36

**Fig 3:**
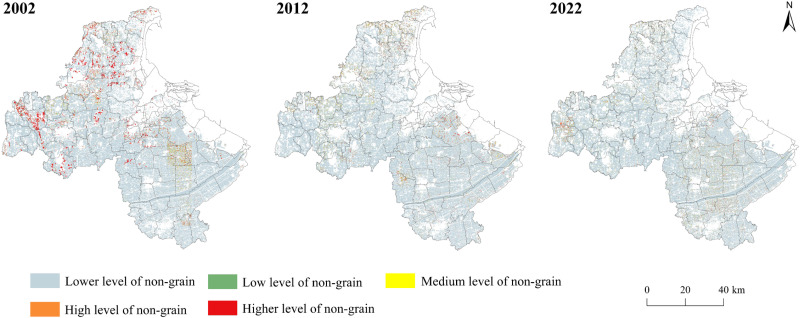
Spatial pattern of cultivated land non-grain in Lianyungang City. Note: Detailed item information is shown in S1 Table.

Cultivated land non-grain level showed strong spatial differentiation in Lianyungang City, was the northwest-southeast high distribution pattern, and there was a tendency to the central and southeastern concentration from 2002 to 2022. Cultivated land non-grain spatial pattern changed significantly from 2002 to 2012. higher degree of non-grain highly shrunk, by the centralized distribution of the western and northern regions of Lianyungang City, the distribution of the fragmented distribution; Medium degree of non-grain by the eastern Tongxing town, Four teams town, Yang set city to the east, southern town clusters spread. The level of non-grain in the northern towns declined, and medium and higher degrees of non-grain spread to the central and southeastern regions from 2012 to 2022. Overall, the lower level of non-grain is transformed into medium and higher level, and the spatial distribution of medium and higher level of non-grain gradually concentrates on the central, eastern, and southern regions. The results of the measurement of cultivated land non-grain level are shown in Fig 3 and S1 Table.

#### 3.1.2 Evolution of cultivated land non-grain structure.

The grain cultivation area of cultivated land showed a continuous downward trend, mainly shifting to non-grain types such as greenhouse vegetable cultivation, construction and development occupation, and abandonment in Lianyungang City from 2002 to 2022 (Tables 3–5). The area of forest planting, aquaculture in pits and ponds, construction and development, and abandonment have all shown an increasing trend. Among them, the area of cultivated land occupied by construction and development and abandoned cultivated land both show a trend of decreasing first and then increasing, and the area of both of them increases more from 2012 to 2022, with an increase of 91.37 km² and 81.46 km², with a dynamic rate of 182.37% and 442.17% respectively. The area of cultivated land occupied by forest plantation and pit-pond aquaculture increased by nearly 10% per year from 2002 to 2012. The forest plantation area decreased slightly from 2012 to 2022, while the area occupied by pit-pond aquaculture continued to increase with a motivation of 28.54 percent. Grain and greenhouse vegetable cultivation areas show a decreasing trend, and the two have a more significant inter-conversion relationship. Among them, the area of grain cultivation decreases at a rate of 0.04% per year, with a more significant decrease (106.80 km²) from 2002 to 2012, and the reduced area is mainly converted into greenhouse vegetable cultivation and is principally converted into greenhouse vegetable cultivation, construction and development, and abandonment of land from 2012 to 2022. The area of greenhouse vegetable cultivation shows an “inverted V” trend, increasing at an annual rate of 6.05 percent from 2002 to 2012 and decreasing at an annual rate of 4.08 percent from 2012 to 2022, with the reduced area mainly shifted to grain cultivation.

**Table 3:** Transfer change of cultivated land non-grain type from 2002 to 2012 (km^2^).

2002	2012						
	Grain cultivation	Greenhouse vegetable cultivation	Forest plantation	Pit-pond aquaculture	Construction	Abandonment	Sum
Grain cultivation	3335.93	271.96	9.00	0.62	3.62	1.32	3622.45
Greenhouse vegetable cultivation	160.56	26.93	0.97	0.014	0.47	0.37	189.31
Forest plantation	3.60	0.94	0.20	0.00	0.31	0.11	5.16
Pit-pond aquaculture	0.20	0.11	0.00	0.012	0.011	0.00	0.33
Construction	9.86	1.76	0.032	0.00	0.55	0.04	12.24
Abandonment	5.49	2.16	0.0018	0.00	0.054	0.0072	7.71
Sum	3515.64	303.86	10.20	0.065	5.01	1.85	

**Table 4:** Transfer change of cultivated land non-grain type from 2012 to 2022 (km^2^).

2012	2022						
	Grain cultivation	Greenhouse vegetable cultivation	Forest plantation	Pit-pond aquaculture	Construction	Abandonment	Sum
Grain cultivation	3194.46	155.58	7.29	2.48	82.21	73.63	3515.65
Greenhouse vegetable cultivation	257.00	22.75	2.14	0.03	13.17	8.78	303.87
Forest plantation	8.55	0.46	0.24	0.00	0.42	0.54	10.21
Pit-pond aquaculture	0.62	0.01	0.00	0.00	0.02	0.01	0.66
Construction	3.71	0.66	0.09	0.00	0.28	0.27	5.01
Abandonment	1.13	0.33	0.00	0.00	0.30	0.08	1.84
Sum	3465.47	179.79	9.76	2.51	96.4	83.31	

**Table 5:** Changes in non-grain structure of cultivated land in Lianyungang City from 2002 to 2022.

Type of land use	2002—2012		2012—2022		2002—2022	
	Transfer area/km²	Dynamic rate/%	Transfer area/km²	Dynamic rate/%	Transfer area/km²	Dynamic rate/%
Grain cultivation	−106.80	−0.29	−50.18	−0.14	−156.98	−0.04
Greenhouse vegetable cultivation	114.55	6.05	−124.07	−4.08	−9.52	−0.05
Forest plantation	5.03	9.75	−0.45	−0.44	4.58	0.42
Pit-pond aquaculture	0.32	9.94	1.86	28.54	2.18	0.78
Construction	−7.24	−5.91	91.37	182.37	84.13	0.79
Abandonment	−5.87	−7.61	81.46	442.17	75.59	0.82

#### 3.1.3 Characteristics of spatial aggregation of cultivated land non-grain.

Based on the Global Moran’s I method of ArcGIS10.8 software to measure the spatial correlation of cultivated land non-grain and test the significance of the measurement results, the results show that they all passed the test of significance level of 0.01 (Table 6). Global Moran’s I show a continuous decreasing trend from 0.90 to 0.42, indicating that the spatial correlation gradually weakens but still presents a more significant spatial correlation from 2002 to 2022.

**Table 6:** Significance test of global autocorrelation index.

Indicator	2002	2012	2022
$Moran^{\prime }sI$	0.8992	0.4899	0.4190
P−value	0.001	0.001	0.001
Z−value	2861.3996	1503.6703	1358.0164

**Fig 4:**
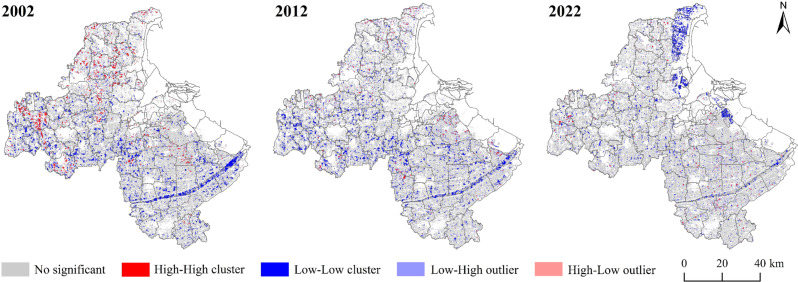
Local spatial correlation map of cultivated land non-grain from 2002 to 2022.

Based on the analysis of global spatial autocorrelation of cultivated land non-grain, local spatial autocorrelation was used to analyze the spatial clustering characteristics of cultivated land non-grain (Fig 4). The spatial differentiation of cultivated land non-grain level was more significant in Lianyungang City from 2002 to 2022. The area of the high-high type is significantly reduced, and it is mainly distributed in the western and northern regions of Lianyungang City, but it has changed over to a multi-center dispersed distribution. The low-high type distribution is less significant. The low-low type is gradually distributed from the southern part of Lianyungang City to the northern coastal area. The high-low type shows more significant dispersion characteristics.

### 3.2 Analysis of driving factors for cultivated land non-grain

#### 3.2.1 Driving factor indicator system construction.

The study selected ten natural and socio-economic factors to detect the driving factors of cultivated land non-grain, and the specific influencing factors are described in Table 7. All the data were unified into CGCS2000 projection, and the DEM data were extracted and processed to obtain the slope by the “SLOPE” tool in ArcMap10.2 software and the resolution of the data was adjusted to 3m by resampling the data of irrigation potential, NDVI, NPP, Precipitation, GDP, and Population density, The “Euclidean distance” in ArcMap 10.2 was used to calculate the Transport accessibility and Distance to the town center.

**Table 7:** Influencing factor index system.

Impact factors	Indicator	Describe
Natural environmental	Altitude(X_1_)	Elevation characteristics of the plot, reflecting the degree of relief of the terrain, were calculated using DEM data
	Slope(X_2_)	Steepness of the plot, calculated using DEM data
	Irrigation potential(X_3_)	The convenience of access to irrigation, characterized using the distance from the plot to the water system
	NDVI(X_4_)	Reflects the state of vegetation cover and growth on the surface.
	NPP(X_5_)	The capacity of photosynthesis in arable systems to fix atmospheric CO_2_
	Precipitation(X_6_)	Reflecting crop growing conditions on cultivated land
Economic and society	GDP(X_7_)	Total GDP per grid, reflecting the socio-economic situation in the study area
	Population density(X_8_)	Number of population distributions per grid, reflecting the demographic profile of the study area
	Transport accessibility(X_9_)	Reflecting the convenience of transport of agricultural products
	Distance to town center(X_10_)	Reflects the convenience of farming agricultural products

#### 3.2.2 Results of OLS analyses.

Non-spatial linear regression simulation was performed using the ordinary regression model (OLS), and the regression coefficients and parameters of the OLS regression model were obtained by combining and testing the above ten influencing factors (Table 8). The variance inflation factor (VIF) is often used to assess local multicollinearity, and the VIF > 7.5 indicates that there is redundancy in the explanatory variables [51]. In the results of this test, the VIF of each explanatory variable is less than 7.5, indicating that the equation variables are reasonable. The test showed that the remaining nine factors, except slope, were all significant at the 1% level, so the differences in the spatial effects of these nine factors on the evolution of cultivated land non-grain were further analyzed.

**Table 8:** Regression coefficient and fitting parameters of OLS model.

Variant	2000		2012		2022	
	*VIF*	P-value	*VIF*	P-value	*VIF*	P-value
X_1_	1.515431	0.001332^*^	1.534098	0.000019^*^	1.218400	0.000275^*^
X_8_	1.030758	0.000003^*^	1.079336	0.000002^*^	1.195315	0.000011^*^
X_4_	1.861422	−0.024375^*^	2.580265	−0.021824^*^	1.785922	−0.010228^*^
X_5_	1.037693	0.026743^*^	1.238164	−0.004622^*^	1.800779	−0.000823^*^
X_9_	1.083331	0.000001^*^	1.059880	0.000001^*^	1.218400	0.000001^*^
X_3_	1.097798	−0.000007^*^	1.079906	−0.000006^*^	1.090393	−0.000002^*^
X_7_	1.100870	0.000020^*^	2.074001	0.000007^*^	1.102630	0.000004^*^
X_6_	1.165132	−0.000053^*^	1.177619	−0.000016^*^	1.268454	−0.000025^*^
X_10_	1.141371	−0.000002^*^	1.143271	0.000000^*^	1.138017	0.000000^*^
R^2^	0.022915		0.005835		0.004648	
VIC_C_	−8805.0458		−42982.8773		−46797.5877	

Precipitation, irrigation potential, and NDVI significantly negatively affected the spatial differentiation of cultivated land non-grain in Lianyungang City, indicating that rainfall, irrigation conditions, and vegetation growth conditions inhibited cultivated land non-grain. The closer the distance to surface water sources, the denser the water network, the more abundant the irrigation water sources, the better the production conditions, the lower the input cost of irrigation facilities, the higher the farmers’ incentive to grow food, and the lower the farmers’ willingness to non-grain. There is a significant positive correlation between transportation, distance from the town center, population density, and spatial differentiation of cultivated land non-grain, and the positive correlation effect of these three factors is more significant. The higher the population density, the more developed the transportation is. The closer the distance from the town center is, the higher the level of urbanization is, the higher the level of economic development is, the more inclined farmers are to cultivate crops with higher value of economic outputs, and the structure of agricultural production tends to be non-grain more often. In addition, elevation and GDP have obvious positive promotion effects on the non-grain cultivated land. The positive promotion or negative inhibition of these factors on the cultivated land non-grain also indicates that the spatial pattern of cultivated land non-grain is subject to the joint action of natural and socio-economic factors in Lianyungang City.

### 3.3 Spatial zoning of cultivated land non-grain

**Fig 5:**
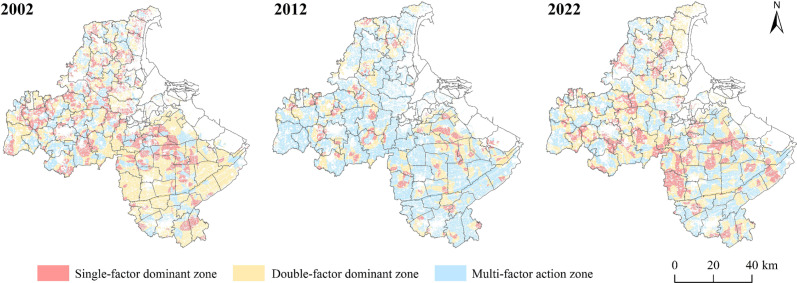
Non-grain space zoning of cultivated land. Note: Detailed item information is shown in S2 Table.

The study further selected GDP, Population density, NDVI, and Precipitation through the GWR model, which has a more significant impact on cultivated land non-grain in Lianyungang City, to detect the influence of these four factors on cultivated land non-grain. According to each detection unit’s dominant factor situation, the cultivated land non-grain was divided into the single-factor dominant, two-factor dominant, and multi-factor action. The results of influencing factors zoning of cultivated land non-grain are shown in Fig 5 and S2 Table.

#### 3.3.1 Single-factor dominant zone.

**Fig 6:**
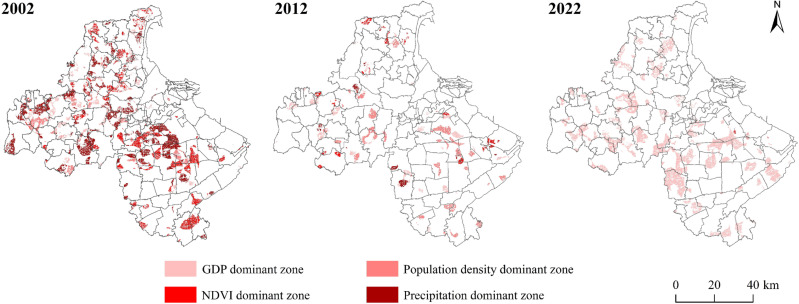
Spatial distribution map of single factor dominant zone.

The area of single-factor dominant zone of cultivated land non-grain showed an overall decreasing trend from 2002 to 2022, which showed a relatively significant decline from 23.05% to 8.45% from 2002 to 2012, and the area of single-factor dominant zone increased from 2012 to 2022, accounting for 19.50% (Fig 6, Table 9). The distribution of the GDP dominant zone is more dispersed, with no apparent aggregation, and the area change is relatively small. The population density dominant zone is mainly distributed in the central and western towns of Lianyungang City, accounting for about 3% from 2002 to 2012, and is a substantial downward trend from 2012 to 2022. NDVI dominant zone decreased from 285.01km^2^ to 1.49 km^2^, accounting for 7.54% to 0.04%, the spatial distribution of the central Lianyungang City by the Four Teams Town, Yangji Town, Bailu Town, and Xinji Town in the south to no apparent distribution. Precipitation dominant zone is significantly reduced, clustered, and distributed in the northwestern region of Lianyungang City, with a small amount scattered in the southern towns and finally dispersed in Nangang town, indicating that the dominant action of precipitation on the cultivated land non-grain is gradually weakened.

**Table 9:** The area and proportion of single factor dominated zone.

Secondary zoning	2002		2012		2022	
	Area/km^2^	Proportion/%	Area/km^2^	Proportion/%	Area/km^2^	Proportion/%
GDP dominant zone	83.89	2.22%	92.92	2.46%	732.94	19.40%
Population density dominant zone	96.72	2.56%	150.09	3.97%	2.44	0.06%
NDVI dominant zone	285.01	7.54%	49.47	1.31%	1.49 1.49	0.04% 0.04%
Precipitation dominant zone	405.40	10.73%	26.82	0.71%	–	–

#### 3.3.2 Double-factor dominant zone.

**Fig 7:**
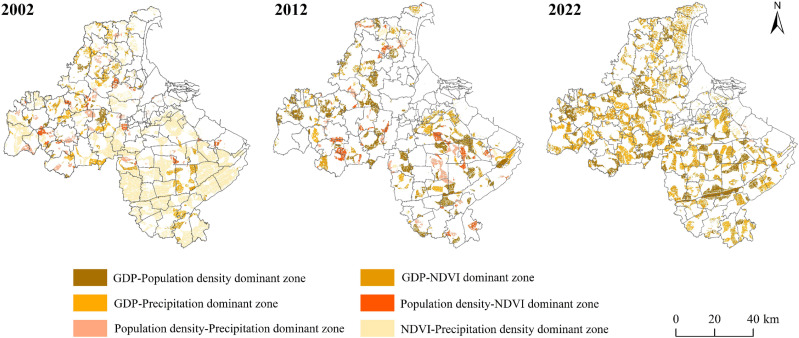
Spatial distribution of double factors dominant zone.

The area of the double-factor dominant zone of cultivated land non-grain decreased overall from 2002 to 2022, of which it significantly reduced from 2002 to 2012, with the area proportion dropping from 52.15% to 25.41%, while it showed a significant increasing trend from 2012 to 2022, with the area share of 42.36% (Fig 7 and Table 10). The area of the GDP-Population density dominant zone showed an upward trend, with the zone concentrated in Baitabu town and Banzhuang town in the north to a uniform distribution in Lianyungang City, which indicates that the degree of influence of cultivated land non-grain by GDP-Population density is gradually increasing. NDVI-Precipitation dominant zone decreased sharply, indicating that the action of natural factors significantly weakened, especially for the south-central of Lianyungang City, where the degree of influence has reduced dramatically.GDP-NDVI, GDP-Precipitation dominant zone increased significantly, and the proportion of GDP-NDVI dominant zone increased from 2012 to 2022, indicating that the positive effect of GDP-NDVI double factors on the cultivated land non-grain continued to improve in Lianyungang City, and the area of double-factors dominated zone of other types decreased to varying degrees from 2002 to 2022.

**Table 10:** The area and proportion of double factors dominant zone.

Secondary zoning	2002		2012		2022	
	Area/km^2^	Proportion/%	Area/km^2^	Proportion/%	Area/km^2^	Proportion/%
GDP-Population density dominant zone	53.57	1.42%	438.65	11.61%	427.61	11.32%
GDP-NDVI dominant zone	141.66	3.75%	169.74	4.49%	839.15	22.21%
GDP-Precipitation dominant zone	188.96	5.00%	90.10	2.38%	326.67	8.65%
Population density-NDVI dominant zone	68.47	1.81%	144.93	3.84%	1.07	0.03%
Population density-Precipitation dominant zone	108.19	2.86%	91.42	2.42%	1.61	0.04%
NDVI-Precipitation dominant zone	1409.46	37.31%	25.30	0.67%	4.44	0.12%

#### 3.3.3 Multi-factor action zone.

**Fig 8:**
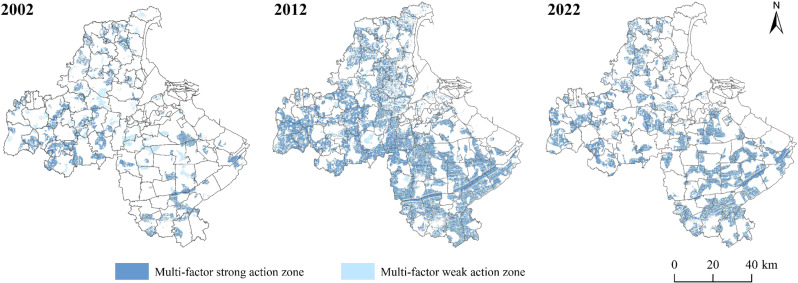
Spatial distribution of multi-factor action zones.

The area of the multi-factor weak action zone of cultivated land non-grain continued to decrease from 2002 to 2022, and the multi-factor strong action zone increased (Fig 8 and Table 11). The multi-factor weak action zone decreases to 205.13 km^2^, and the proportion decreases from 5.46% to 0.03%. Multi-factor strong action zone is mainly distributed in the northwestern and southern towns of Lianyungang City, increasing 1,721.07km^2^ from 2002 to 2012, accounting for an increase from 19.34% to 64.89%, indicating that the cultivated land non-grain is subject to a robust joint action of multi-factors in Lianyungang City. The area of the multi-factors’ strong action zone decreased significantly from 2012 to 2022, indicating that the multi-factors on cultivated land non-grain are weakened while the influence of single- or double-factor dominant action is enhanced.

**Table 11:** The area and proportion of the multi-factor action zone.

Secondary zoning	2002		2012		2022	
	Area/km^2^	Proportion/%	Area/km^2^	Proportion/%	Area/km^2^	Proportion/%
Multi-factor weak zone	206.23	5.46%	47.08	1.25%	1.10	0.03%
Multi-factor strong zone	730.53	19.34%	2451.60	64.89%	1439.59	38.10%

## 4 Discussion and conclusion

### 4.1 Discussion

#### 4.1.1 Driving mechanism of cultivated land non-grain.

Cultivated land non-grain is not only caused by a single factor but is the result of multi-factors action in Lianyungang City, which is consistent with the results of previous studies [18,52], and the multi-factors mainly include two aspects of natural and socio-economic factors.

Natural factors such as elevation are the main reasons for converting cultivated land to forest plantation [53–55], and the outflow of labor and differences in planting efficiency are the underlying reasons. Firstly, the cultivated land in higher elevation converted to forest plantation is higher due to the unsuitability of growing food [56]. Secondly, the decline of the rural resident population in Lianyungang City and the flow of many rural laborers to the cities have prompted some farmers to plant forests with fewer inputs, and some marginalized cultivated land has been left fallow [57], a phenomenon that is more prevalent in mountainous areas, which is more in line with the results of the study by Wu on the influencing factors of cultivated land non-grain in the mountainous areas of northern Guangdong Province [58]. Steep slopes, fragmentation, poor quality, and other cultivated land with poor suitability for grain cultivation make it challenging to improve grain cultivation’s benefits through land remediation, so farmers often choose to plant non-grain crops with higher economic benefits [59].

The economic radiation of the central urban area directly affects the cultivated land non-grain. The economic development of the central urban area often leads to the cultivated land non-grain use in the neighboring districts and counties because the cost of planting food around the central urban area is often higher, and thus, the farmers’ willingness to plant food is also relatively low [17,60]. Also, limited by farmers’ cognition and market sensitivity, farmers’ planting behaviors are easily influenced by other farmers’ planting behaviors, leading to non-grain use of cultivated land [61,62].

Irrigation potential is the main reason for the conversion of cultivated land from food to vegetable and forest cultivation, and the distance factor is the root cause of the influence of cultivated land non-grain, which is similar to the results of the study by He on the most important drivers of cultivated land non-grain in karst troughs and valleys [63]. The high water demand for grain crops compared to cash crops such as vegetables and fruits, coupled with the cost of irrigation facilities, persistently low grain prices, and the yearly increase in irrigation costs, have led to a continuous decline in the incentives of farmers, and farmers will rationally choose to grow crops with higher economic value [64–66], which also reflects the influence of the economy on the cultivated land non-grain. In addition, the degree of transport convenience and distance from settlements also affect cultivated land use. Areas with convenient transportation are conducive to the large-scale and mechanized use of cultivated land, and farmers are more motivated to grow food. Areas with inconvenient transportation are often due to accelerated urbanization and industrialization. Many working people in the countryside migrate to the areas with better economic development, and the remaining number of aged and weakened farmers, who are limited by cultivation technology and labor, tend to prefer cultivating labor-saving and simple vegetables and forests [20], which is more consistent with the results of many scholars’ studies on the transformation of cultivated land in mountainous areas [67].

This study better reflects the characteristics of the evolution of cultivated land non-grain in Lianyungang City, partitions the spatial differentiation of the evolution of cultivated land non-grain based on the influencing factors, and then proposes a differentiated strategy for the control of cultivated land non-grain. But only focuses on the apparent non-grain caused by land use changes, and the broad concept of food crops and invisible non-grain is still worth further exploration. Due to the limitation of data acquisition and its completeness, the driving factors of cultivated land non-grain are only considered to be some natural, economic, and social factors, and it failed to detect different types of non-grain on cultivated land separately. The structure of the regional agricultural management bodies and related policies also affect the cultivated land non-grain. Hence, future research on the driving factors needs to start from multiple perspectives and elements and combine with questionnaire surveys and field research to conduct more in-depth discussions.

#### 4.1.2 Control strategy of cultivated land non-grain zone.

Aiming at the phenomenon of cultivated land non-grain that prevails in the current main grain-producing areas, this study develops control measures more in line with the spatial zoning of cultivated land non-grain based on the influencing factors of the GWR model in each zoning area.

(1) Single-factor dominant zone should enhance the synergistic governance capacity of relevant subjects of cultivated land use [68]. Fully understand the complexity of multi-temporal and multi-subjects in cultivated land use and effectively carry out cultivated land non-grain governance actions. First, the agricultural subsidy system should be improved to protect farmers’ fundamental rights and interests. From the perspective of planting varieties and their input costs, give full play to the leading action of local governments, open up food outlets through multiple channels, enhance the benefits of grain cultivation, and ensure that permanent basic farmland is used for grain cultivation. In addition, it should also implement subsidies for grain cultivation to households raise the subsidy standard, and adopt various methods to enhance farmers’ incentive to grow grain. Secondly, accelerating the cultivation of new agricultural management bodies, promoting land transfer by activating land management rights, realizing the concentration of cultivated land management, the moderate scale of agricultural production, and reducing the impact of the marginalization of cultivated land and the aging and weakening of the agricultural population on the cultivated land non-grain. Thirdly, the benefits of grain cultivation in different terrain conditions have an impact on farmers’ operation and planting choices. Therefore, agricultural land improvement measures should be arranged based on varying terrain slopes, soil conditions, irrigation potential, and other zoning arrangements to use regional natural conditions fully.
(2) The double-factor dominated zone must strengthen the government’s regulation and optimization guidance. Government intervention is mandatory and guides agricultural operations [69], directly affecting cultivated land use development direction. First, to carry out a replanting assessment of non-grain plots that can be resumed for grain cultivation, to assign a replanting cost index to each non-grain plot, to select the optimal replanting method and sequence based on the score, to reduce the stock of non-grain plots of cultivated land, and to promote the large-scale operation of cultivated land. Secondly, differentiated cultivated land use control measures have been formulated for areas with different dominant factors, crops on permanent bare farmland have been classified and supervised using film-guard enforcement, and a monitoring mechanism for cultivated land crop types has been established. Thirdly, land leveling and road construction projects have been promoted to improve irrigation conditions on farmland and to integrate fragmented cultivated land due to the fragmentation of cultivated land tenure to encourage the upgrading of cultivated land quality.
(3) Multi-factor action zone should promote synergistic enhancement of the elements of the cultivated land use system [70,71]. First, based on a significant food perspective, a rational layout based on the principles of grain for grain, economy for the economy, and forest for the forest determines the direction of land use that is not suitable for grain and ensures that the management of cultivated land non-grain follows the laws of the system, to implement measures according to the local situation. Second, the agricultural insurance system can be improved by expanding the variety and scale of agricultural insurance and increasing the amount of agricultural insurance subsidies. At the same time, it should enhance agricultural talent incentive policies, safeguard professional and technical talent positions, and form a complete chain of teams throughout all aspects of agricultural production.

### 4.2 Conclusion

The study takes Lianyungang City in Jiangsu Province as an example, integrates techniques such as high-fraction remote sensing imagery and spatial analysis to analyze the evolutionary characteristics of cultivated land non-grain from 2002 to 2022, explore the driving mechanisms of its evolution, and formulate zonal regulation strategy. The main conclusions are as follows:

(1) The level of cultivated land non-grain in Lianyungang City showed an upward trend from 2002 to 2022, and grain cultivation mainly shifted to greenhouse vegetables, construction and development occupation, and abandonment. Cultivated land non-grain increased from 6.01% to 11.10%, with a more significant increase from 2002 to 2012. The conversion of cultivated land for grain and greenhouse vegetable cultivation has been more critical, and the area of non-cultivated land has changed more drastically, with the area of greenhouse vegetable cultivation decreasing and the rest of the types increasing, indicating that the phenomenon of non-food cultivation of cultivated land has continued to deepen and that there is a clear trend towards non-food cultivation.
(2) The level of cultivated land non-grain showed a high distribution pattern from northwest to southeast, and the distribution of cultivated land non-grain had firm heterogeneity. The spatial pattern of non-grain cultivated land gradually weakened, and Moran’s I decreased from 0.90 to 0.42, indicating that its spatial correlation was slowly weakening. The degree of cultivated land non-grain transformed from low to medium and higher, and the spatial distribution gradually concentrated in the central, eastern, and southern regions, indicating that there is a tendency for further spreading of cultivated land non-grain.
(3) The evolution of cultivated land non-grain was a complex result of the joint action of natural and socio-economic factors. The dominant factors of the spatial differentiation of cultivated land non-grain were different in different periods, among which GDP, population density, NDVI, and precipitation were always the main influencing factors, and GDP and population density reflected the regional endowment of farm resources and labor resources, which together with NDVI and precipitation formed different combinations that affected cultivated land non-grain, and the main driving factors in other regions such as mountainous areas and karst troughs and valleys have certain differences and similarities.
(4) The evolution of cultivated land non-grain was divided into single-factor dominant, double-factor dominant, and multi-factor action, in which the single-factor dominant and double-factor dominant action were weakened, and the multi-factor action was strengthened. It reveals that the future governance of cultivated land non-grain should have a holistic view, and comprehensively consider a variety of influencing factors in Lianyungang City, Further, based on the regional characteristics of the main synergy, government regulation, and the enhancement of system elements, this study proposed a zoning governance strategy that can effectively improve the non-grain governance level of cultivated land.

## Supporting information

S1 Table Changes in the area of level of cultivated land non-grain. (XLSX)

S2 Table Changes in the area and proportion of influencing factors zoning of cultivated land non-grain. (XLSX)
